# Comparison of the prognosis and recurrence of apparent early-stage ovarian tumors treated with laparoscopy and laparotomy: a meta-analysis of clinical studies

**DOI:** 10.1186/s12885-015-1604-3

**Published:** 2015-08-26

**Authors:** Ying Zhang, Shuying Fan, Yang Xiang, Hua Duan, Li Sun

**Affiliations:** 1 Department of Gynecology Minimally Invasive Center, Beijing Obstetrics and Gynecology Hospital,Capital Medical University, #17 Qi He Lou Street, Dongcheng District, Beijing, 100006 China; 2 Department of Gynecology and Obstetrics, Kailuan General Hospital, #57 Xinhua East Road, Tangshan, 063000 Hebei China; 3 Department of Obsteric and Gynecology, Peking Union Medical College Hospital, Peking Union Medical College, Chinese Academy of Medical Sciences, #1 Shuai Fu Yuanx, Dong Cheng District, Beijing, 100730 China; 4 Department of Medical Oncology, the Central Hospital of Xuzhou, the Cancer Institute of Southeast University, Xuzhou, Jiangsu 221009 China

## Abstract

**Background:**

This meta-analysis aimed to evaluate the prognosis and recurrence of apparent early-stage ovarian tumors treated with laparoscopy compared with laparotomy.

**Methods:**

Clinical studies published in English were retrieved from the computerized databases Medline and Embase. A meta-analysis was performed to investigate the differences in the efficacy and safety of laparoscopy versus laparotomy in terms of postoperative complications, lengths of hospital stay, recurrence rates, and disease-free survival times using the random effects model. The studies were independently reviewed by two investigators. Data from the eligible studies were extracted, and the meta-analysis was performed using the Comprehensive Meta-Analysis program, version 2 (CMA-2; Biostat, Englewood, NJ, USA).

**Results:**

A total of 8 studies were included in the analysis. The results showed that laparoscopic surgery was significantly associated with lower rates of complications (OR = 0.433, *P* = 0.019) and shorter postoperative hospital stays (weighted mean difference [WMD] = −0.974, *P* < 0.001). There was no significant difference in the rates of recurrence (OR = 0.707, *P* = 0.521) between patients with apparent early-stage ovarian tumors who were treated using laparoscopy and those who underwent laparotomy. No publication bias was detected.

**Conclusions:**

Laparoscopic surgery shows favorable prognostic outcomes in terms of postoperative complication rates and postoperative hospital stay durations. Further studies with longer follow-up periods are required to confirm recurrence and survival outcomes after laparoscopic surgery in patients with apparent early-stage ovarian tumors.

## Background

Ovarian cancer is among the major gynecological malignant tumors, and it ranks first in mortality among gynecological malignancies. Studies have shown that the 5-year survival rate for ovarian cancer is as low as approximately 30 %, though these rates have markedly increased in recent decades with the development of new treatments and regimens [1, 2].

Ovarian cancer is difficult to identify in its early stage, and 70 % of patients are diagnosed at an advanced stage, resulting in a poor prognosis. Indeed, the early diagnosis of ovarian cancer is crucial to improving treatment efficacy. Currently, the standard treatment for early-stage ovarian cancer is primarily surgical management (with or without chemotherapy). According to the International Federation of Gynecology and Obstetrics (FIGO) guidelines, the optimal staging procedures for ovarian cancer are complete abdominal hysterectomy, bilateral salpingo-oophorectomy, peritoneal biopsy, omentectomy, diaphragmatic scraping, bilateral pelvic and para-aortic lymph node dissection, and maximal debulking efforts to leave “no visible and no palpable disease” [3]. Clinical practice has proven that laparotomy is effective as a traditional surgical treatment for ovarian cancer [4, 5]. In addition, the efficacy of laparoscopy, a minimally invasive procedure, has been demonstrated in recent years [6]. Laparoscopy offers the primary advantages of minimal trauma and rapid recovery and is currently widely used in the diagnosis and treatment of malignant gynecological tumors. Studies suggest that compared with laparotomy, laparoscopy is associated with shorter hospital stays, lower morbidity, and shorter recovery times [7, 8].

Nonetheless, studies examining the effects of laparoscopy versus laparotomy in treating apparent early-stage ovarian cancer have involved limited numbers of patients, and randomized controlled trials are not available. The present review systematically combines existing clinical studies that compared the effects of laparoscopy versus laparotomy in treating apparent early-stage ovarian cancer to evaluate the prognosis and recurrence of laparoscopy and reach a conclusion with high credibility. A random-effects meta-analysis following the MOOSE guidelines [9] for observational studies and the QUORUM guidelines for randomized controlled trials was utilized [10].

## Methods

### Search strategy for identifying studies

An in-depth literature search was performed using the keywords “laparoscopy,” “ovarian tumor,” “clinical study,” and “early-stage” in various combinations. The computerized databases PubMed (from 1980 to May 2014) and Embase (from 1980 to May 2014) were searched to identify clinical studies in English-language journals. We also searched the related references in the retrieved studies and reviewed articles from the bibliographic database. The corresponding authors of some studies were contacted for information beyond what was available in their published articles.

### Article selection criteria

All clinical studies that explored the differences in prognosis and/or recurrence of apparent early-stage ovarian tumors (stage I and stage II, according to the FIGO classification) treated with laparotomy versus laparoscopy were considered eligible for the analysis. Two investigators (Ying Zhang and Hua Duan) independently assessed the articles for relevance. Articles were excluded if (1) no comparisons were made between laparoscopy and laparotomy and (2) no standardized effect size could be calculated. This study was approved by the Institutional Review Board of the Beijing Obstetrics and Gynecology Hospital affiliated with the Capital University of Medical Sciences. All of the procedures used in this study are in compliance with the Helsinki Declaration.

### Statistical analyses

Data management and analysis were performed using the Comprehensive Meta-Analysis program, version 2 (CMA-2; Biostat, Englewood, NJ, USA). The outcomes were pooled statistically using the event rates calculated for postoperative complications and recurrence rates and the standard mean difference for length of hospital stay. A random-effects meta-analysis was conducted to investigate the efficacy and safety of laparoscopy versus laparotomy to treat apparent early-stage ovarian tumors.

The measure of heterogeneity was evaluated using the Cochran Q test. Additionally, heterogeneity (measured as I^2^) was used to assess the percentage of the total variation from all studies. A high value for I^2^ indicates heterogeneity. Publication bias was evaluated using Egger’s test.

## Results

**Fig. 1:**
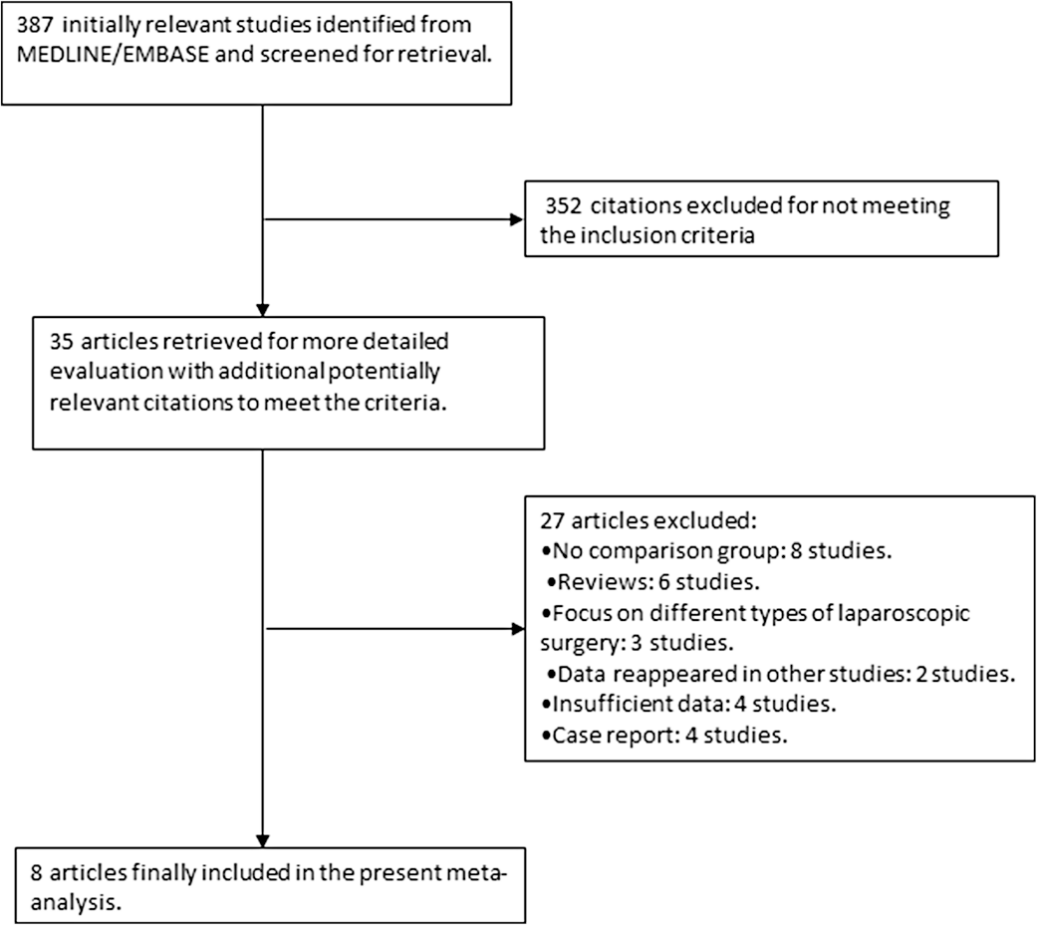
Flow chart describing the article selection process

A total of 387 abstracts were initially selected through database searches, and 352 articles were excluded because they failed to meet the inclusion criteria. Of the remaining 35 articles, 8 did not have a comparison group, 6 were review articles, 3 focused on different types of laparoscopic surgery, 2 included the same data that were presented in other studies, 4 did not provide sufficient information to calculate an effect size, and 4 were case studies. The articles excluded from this study are shown in Fig. 1, and the 8 articles [8, 11–17] selected for our analysis are shown in Table 1.

**Table 1:** Main characteristics of the selected studies

Citation	Mean patient age (years)	Number of patients	Body mass index	Upstaging rate	Duration of operation (min)		Intraoperative blood loss (ml)		Hospital stay (days)		Number of pelvic lymph node dissections		Number of para-aortic lymph node dissections	
					Laparoscopy	Laparotomy	Laparoscopy	Laparotomy	Laparoscopy	Laparotomy	Laparoscopy	Laparotomy	Laparoscopy	Laparotomy
Liu 2014	50.9	75	NA	20.0 %	209.71 ± 17.57	200.50 ± 20.62	197.14 ± 98.48	345.00 ± 165.95	16.3 ± 6.2	21.9 ± 4.9	18.23 ± 3.27	19.03 ± 3.15	NA	NA
Koo 2013	47.6	77	23.5	29.9 %	192.9 ± 73.5	224.1 ± 85.4	697.9 ± 396.9	972.6 ± 827.8	13.7 ± 5.4	13.1 ± 4.1	26.8 ± 8.5	27.8 ± 13.2	17.7 ± 10.1	21.2 ± 11.2
Lee 2011	43.9	113	23.0	26.5 %	227.6 ± 105.8	184.6 ± 61.4	230.4 ± 183.6	474.8 ± 329.2	6.4 ± 2.6	12.4 ± 5.5	23.5 ± 9.3	22.8 ± 10.2	9.9 ± 7.4	4.8 ± 4.1
Park 2008 _(1)_	44.9	52	22.9	21.2 %	220.7 ± 82.7	274.7 ± 63.2	240.0 ± 228.3	568.2 ± 451.7	8.9 ± 6.1	14.5 ± 5.6	27.2 ± 9.7	33.9 ± 14.5	6.6 ± 6.2	8.8 ± 8.1
Park 2008 _(2)_	46.2	36	23.5	19.4 %	303.8 ± 84.9	290.4 ± 120.8	231.2 ± 117.9	505.3 ± 279.8	9.4 ± 4.1	14.1 ± 4.2	13.7 ± 5.6	19.3 ± 10.1	8.9 ± 7.1	6.4 ± 3.9
Ghezzi 2007	58.4	34	24.9	29.45	377.0 ± 47.0	272.0 ± 81.0	250.0 (50–1,000)	400 (150–1,000)	3.0 ± 2.5	7.0 ± 2.5	25.2 ± 9.3	25.1 ± 5.8	6.5 ± 3.9	7.0 ± 4.5
Lécuru 2006	49.2	148	NA	19.0 %	NA	NA	NA	NA	NA	NA	NA	NA	NA	NA
Chi 2005	49.0	50	25.1	NA	321.0 ± 64.0	276.0 ± 68.0	235.0 ± 138.0	367.0 ± 208.0	3.1 ± 0.7	5.8 ± 2.6	12.3 ± 4.9	14.7 ± 5.7	6.7 ± 2.5	9.2 ± 5.0

(1) and (2) represent different studies by the same first author

**Fig. 2:**
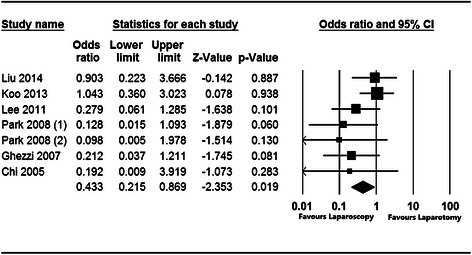
Forest plot for the aggregated rate of laparoscopy-related complications in patients with apparent early-stage ovarian tumors

**Fig. 3:**
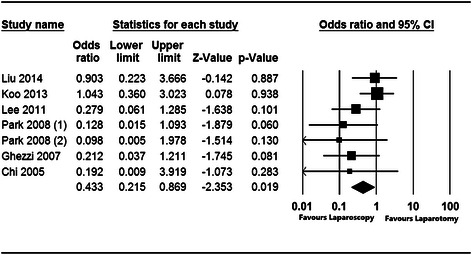
Forest plot for the aggregated length of hospital stays following laparoscopy in patients with apparent early-stage ovarian tumors

**Fig. 4:**
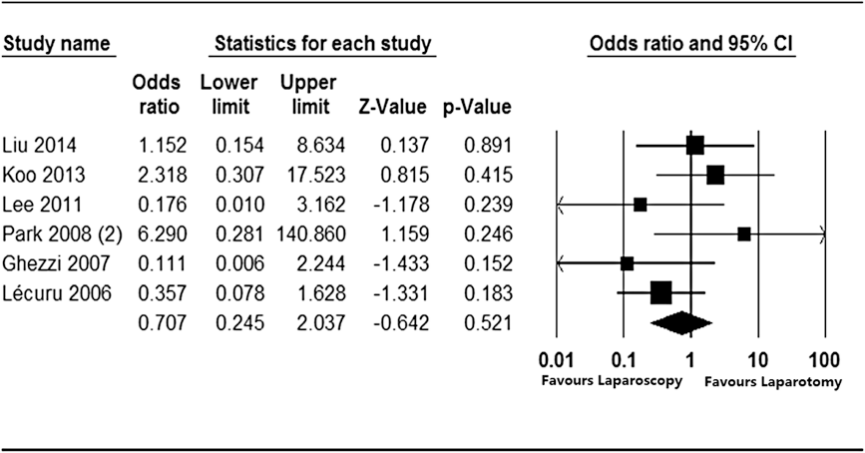
Forest plot for the aggregated laparoscopy recurrence rate in patients with apparent early-stage ovarian tumors

In the 8 studies that were analyzed, the efficacy and safety of laparoscopy versus laparotomy in the treatment of apparent early-stage ovarian cancer were investigated. The pooled clinical studies that examined the prognosis and recurrence of apparent early-stage ovarian tumors treated with laparoscopy showed that compared with laparotomy, laparoscopic surgery was significantly associated with lower complication rates (OR = 0.433, 95 % CI: 0.215 to 0.869, Z = −2.353, *P* = 0.019; Fig. 2) and shorter postoperative hospital stays (WMD = −0.974, SE = 0.220, Z = −4.420, *P* < 0.001; Fig. 3). In terms of recurrence rates, there was no significant difference (OR = 0.707, 95 % CI: 0.245 to 2.037, Z = −0.642, *P* = 0.521; Fig. 4) between patients with apparent early-stage ovarian tumors who were treated with laparoscopy and those who underwent laparotomy. In Liu’s study (2014), the disease-free survival times were 54.3 months and 57.2 months for patients who were treated with laparoscopy and laparotomy, respectively.

There was statistically significant heterogeneity in the models for hospital stay (Q = 24.055, *P* = 0.001, I^2^ = 75.057), but no significant heterogeneity was observed in the models for the rates of complications (Q = 7.041, *P* = 0.317, I^2^ = 14.784) or recurrence (Q = 6.570, *P* = 0.255, I^2^ = 23.898). Publication bias was examined using funnel plots and Egger’s regression test, and both indicated that there was no significant publication bias (*P* > 0.05) in the outcomes of this meta-analysis. Consequently, unpublished data were not further evaluated.

## Discussion

In gynecologic oncology, laparoscopic surgery is considered capable of potentially providing a sufficient degree of visualization, via optical magnification, for allowing for optimal performance and accurate verification of resection, while simultaneously allowing for preservation of vital structures, such as small vessels, that may be barely visible to the naked eye [18, 19]. This study aimed to explore the effectiveness of laparoscopy versus laparotomy for the treatment of apparent early-stage ovarian tumors. The results confirmed the favorable prognostic outcomes of laparoscopy for reducing the lengths of hospital stays and the rates of postoperative complications in patients with apparent early-stage ovarian tumors. Specifically, the aggregated effect size revealed that laparoscopic surgery was significantly associated with fewer complications (OR = 0.433, 95 % CI: 0.215 to 0.869, Z = −2.353, *P* = 0.019) and shorter postoperative hospital stays (WMD = −0.974, SE = 0.220, Z = −4.420, *P* < 0.001). These findings are consistent with those from studies reporting the advantages of laparoscopy in treating ovarian tumors [7, 12, 20]. Lee [12] reported that complete surgical staging via laparoscopy (*n* = 26) resulted in reduced blood loss, earlier diet resumption, lower postoperative pain scores, and shorter hospital stays compared with staging via laparotomy (*n* = 113) in patients with apparent early-stage ovarian cancer. Laparoscopic surgery has been associated with less intraoperative blood loss and shorter postoperative hospital stays compared with laparotomy [17, 21]. A major concern regarding laparoscopic surgery is the risk of port-site metastasis, which has incidence rates of 1 %–16 % [13]. However, in a number of studies on apparent early-stage ovarian cancer, no cases of port-site metastasis or recurrence were reported in patients who had undergone laparoscopy [14, 22]. Additionally, the inability to utilize fine tactile assessment during laparoscopic surgery to assess the extent of disease may result in the non-recognition of occult metastatic deposits of disease that may be situated within difficult to visualize areas within the abdomen and pelvis. To compensate for this deficiency in the use of laparoscopic surgery for staging, we suggest that preoperative examinations, such as PET-CTs, be used to detect early metastases so that they can be resected in a timely manner. Notably, it is impossible to summarize the hospital stay outcomes in this meta-analysis because of the studies’ considerable heterogeneity, which could have resulted from specific differences in the patients’ conditions and the study designs [20, 23, 24].

In this meta-analysis, no significant difference was detected in the recurrence rates (OR = 0.707, 95 % CI: 0.245 to 2.037, Z = −0.642, *P* = 0.521) of patients with apparent early-stage ovarian tumors who were treated laparoscopically and those who underwent laparotomy. Koo [13] conducted a prospective study with a mean follow-up period of 31 months and found that tumor recurrence occurred in 2 (8.3 %) patients in the laparoscopy group and 2 (3.8 %) in the laparotomy group (*P* = 0.585). There was no significant difference in the mean disease-free survival time, which was excellent in both groups (59 months after laparoscopy versus 66 months after laparotomy, *P* = 0.367). Studies have reported that laparoscopy decreases surgical morbidity and improves cancer-related survival times by preserving patients’ cellular immunity [25–27]. In this study, the pooled disease-free survival time was not computed because the related data were only available in two of the included studies (Koo 2013 & Lee 2011). Koo (2013) reported that laparoscopy and laparotomy were associated with disease-free survival times of 59.3 ± 3.78 months and 66.3 ± 1.92 months, respectively. In contrast, Lee (2011) found that patients with apparent early-stage ovarian tumors who received laparoscopy or laparotomy had disease-free survival times of 13.3 ± 10.2 months and 25.7 ± 15.0 months, respectively. However, in a large series that included the laparoscopic staging of 300 patients with apparent early-stage ovarian cancers, laparoscopic surgical management exhibited excellent safety in terms of recurrence and death from disease; 25 patients (8.3 %) underwent immediate laparoscopic staging and 10 (3.3 %) underwent delayed laparoscopic staging, and the 3-year disease free survival and overall survival rates were 85.1 % and 93.6 %, respectively, for all patients [28]. Future research should include more studies with relatively longer follow-ups to investigate recurrence rates and survival outcomes.

The current review retrieved clinical studies published through May 2014 that estimated the prognostic outcomes of laparoscopic treatment for apparent early-stage ovarian tumors. The findings of our review and meta-analysis are therefore valuable for physicians and policy makers, given the benefits of laparoscopic treatment in terms of reducing hospital stays and complication rates for patients with early-stage ovarian tumors. We employed random-effects models based on the heterogeneity of the true effects distribution, which avoided the bias of overstating the precision of findings in fixed-effects models. A limitation of the current study is the small number of studies and the limited numbers of participants involved. This reflects the paucity of high-quality clinical trials that address the efficacy of laparoscopic surgery for treating ovarian tumors. Generalizations of this study’s conclusions to all patients with early-stage ovarian tumors should be considered with caution, and there is still considerable need for higher quality studies with relatively larger sample sizes to address this topic.

## Conclusion

In conclusion, this meta-analysis confirms that laparoscopy has favorable prognostic outcomes in terms of the postoperative complications rate and the lengths of post-operative hospital compared with conventional laparotomy in the treatment of apparent early-stage ovarian tumors. Laparoscopic surgery may be effective and feasible for treating apparent early-stage ovarian tumors.

## Abbreviations

FIGO: the International Federation of Gynecology and Obstetrics
CMA-2: Comprehensive Meta-Analysis Program, version 2
